# Laparoscopic Repair of a Sciatic Hernia Using a Self-Fixating Mesh: A Case Report

**DOI:** 10.70352/scrj.cr.25-0767

**Published:** 2026-02-18

**Authors:** Kimihiro Hattori, Shota Nakashima, Rintaro Sakamoto, Bei Wang, Machi Mizuno, Takuji Sakuratani, Hiroomi Ikeshoji, Noriaki Kojima, Kimitoshi Nishio, Tatsumi Iida, Takao Takahashi

**Affiliations:** Department of Surgery, Gifu Seino Medical Center, Seino Kousei Hospital, Ibi-gun, Gifu, Japan

**Keywords:** sciatic hernia, greater sciatic hernia, laparoscopic surgery, elective surgery, self-fixating mesh, flat mesh, flat-type mesh, mesh repair

## Abstract

**INTRODUCTION:**

A hernia that protrudes through the sciatic foramen is called a sciatic hernia and is classified as a pelvic hernia, although it is the rarest type. We aimed to report a case of elective laparoscopic repair of a sciatic hernia using a self-fixating mesh.

**CASE PRESENTATION:**

The patient was a 78-year-old female who had experienced intermittent numbness in the right thigh for approximately 1 year. Whole-body CT performed during hospitalization for pneumonia revealed small-bowel herniation through the right sciatic foramen. MRI showed the small-bowel protruding above the sacrospinous ligament, confirming diagnosis of a right greater sciatic foramen hernia. Her thigh numbness was attributed to the hernia, and because she had no signs of bowel obstruction or ischemia, elective surgery was planned after pneumonia treatment. Laparoscopic repair involved dissecting the preperitoneal space around the sciatic foramen and placing a self-fixating mesh.

**CONCLUSIONS:**

We ensured a safe operative field and performed careful dissection and non-fixation mesh placement while preserving the vessels, nerves, and ureter around the sciatic foramen, resulting in an uneventful postoperative course. Reports of laparoscopic mesh repair for sciatic hernia remain limited. Based on previously prior cases, we discuss mesh selection, the need for mesh fixation, and the optimal extent of dissection required for placing a sheet-type mesh.

## INTRODUCTION

Sciatic hernia is a type of pelvic hernia and is considered to be the least common among them. Reports on its management are limited, and no standardized surgical approach has been established. Both transabdominal and transgluteal approaches have been described, with most published cases favoring the transabdominal route. Open repair has traditionally been used; however, recent reports describe elective laparoscopic repair in patients without bowel obstruction or ischemic changes in the herniated organ. Various techniques for closing the hernia orifice have been reported, including mesh repair, autologous tissue repair, and primary suture closure; however, no consensus exists on the optimal method. Here, we aimed to report a case of sciatic hernia with small-bowel herniation successfully treated by elective laparoscopic repair using a self-fixating mesh, and to provide a brief review of the relevant literature.

## CASE PRESENTATION

A 78-year-old woman with type 2 diabetes mellitus, hyperlipidemia, hypertension, and no history of abdominal surgery was hospitalized for pneumonia. Whole-body CT performed during this admission incidentally revealed small bowel herniation through the right sciatic foramen, leading to diagnosis of a sciatic hernia. As there were no findings suggesting bowel obstruction or ischemia of the herniated bowel, she was referred to our department after completing pneumonia treatment. On initial examination, she was 152 cm tall and weighed 33.4 kg (body mass index: 14.46 kg/m^2^). Her abdomen was flat and soft, with no tenderness. Laboratory testing at the first visit revealed no significant abnormalities.

### Investigations

**Fig. 1:**
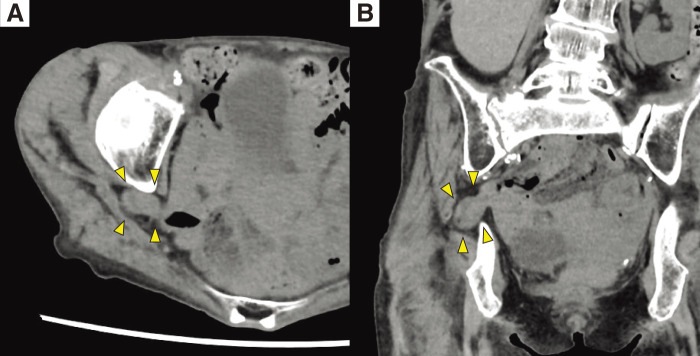
CT findings. (**A**) Axial view. (**B**) Coronal view. Abdominal CT revealed the small-bowel herniated through the sciatic foramen (arrowheads), with no evidence of bowel obstruction or ischemia.

**Fig. 2:**
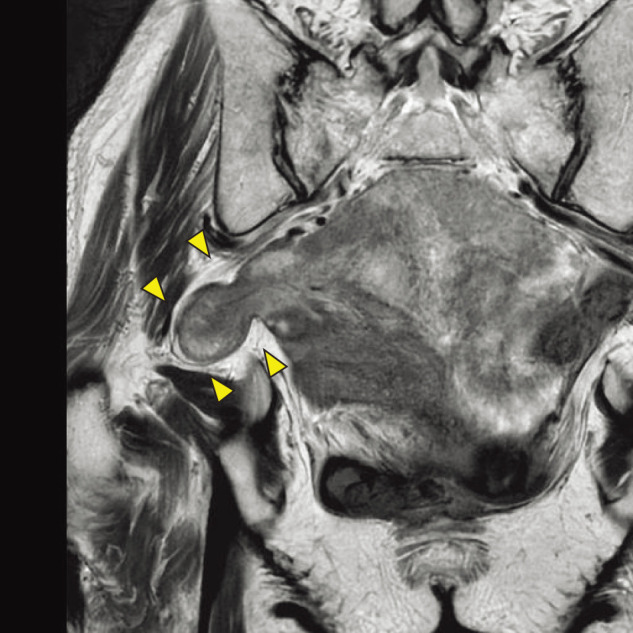
MRI findings. MRI showed a hernia orifice cranial to the sacrospinous ligament, consistent with a greater sciatic foramen hernia (arrowheads).

Abdominal CT revealed small bowel herniation through the right sciatic foramen into the pelvic cavity, leading to a diagnosis of sciatic hernia (**Fig. 1A** and **1B**). There was no small-bowel dilatation or evidence of obstruction. No ascites, mesenteric edema, or other findings suggesting ischemia were observed. Pelvic MRI demonstrated the small bowel protruding above the sacrospinous ligament, consistent with a greater sciatic foramen hernia (**Fig. 2**). Based on these findings, a right greater sciatic foramen hernia with small-bowel herniation was diagnosed. She had no abdominal symptoms, and the absence of bowel obstruction or ischemic changes indicated that emergency surgery was unnecessary. However, she had recurrent numbness in the right thigh, which was attributed to sciatic nerve compression caused by the herniation. Elective surgery was therefore considered appropriate. As there was no small-bowel dilatation and sufficient preoperative evaluation was possible, laparoscopic repair was planned.

### Surgical findings

**Fig. 3:**
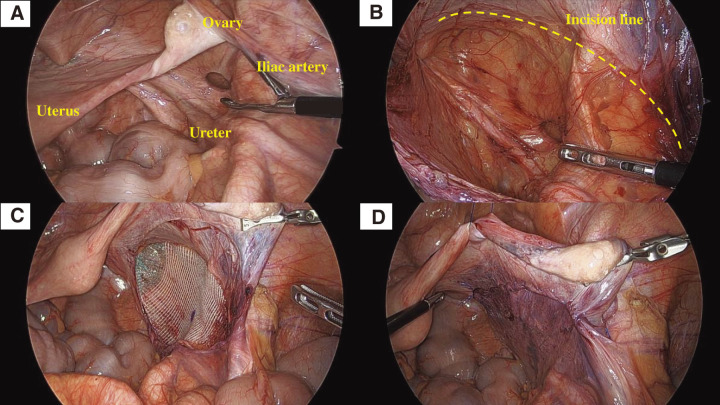
Operative findings. (**A**) A hernia orifice was identified between the right ureter and the infundibulopelvic ligament, and the herniated small bowel had spontaneously reduced. (**B**) The peritoneum was dissected carefully to avoid injury to the surrounding tissues (the yellow dotted line), and a 3-cm margin of dissection was secured around the hernia orifice. The sciatic nerve is not visible because the adipose tissue surrounding the hernia orifice was intentionally preserved to avoid nerve injury. (**C**) A self-fixating mesh was trimmed to fit the dissected area and placed over the orifice, without any additional fixation. A circumferential margin of approximately 3 cm was secured. The mesh was trimmed to approximately 7 × 14 cm. (**D**) The incised peritoneum was closed with a continuous suture, and an adhesion barrier was applied over the dissected area.

Under general anesthesia, the patient was placed in the lithotomy position with a pillow under the lower back to elevate the pelvis. A 12-mm port was inserted at the umbilicus, and two 5-mm ports were placed in the right and left lateral abdominal walls, initiating the procedure with a three-port technique. With the patient in the steep Trendelenburg position, exploration of the pelvic cavity revealed that the herniated small bowel had already reduced spontaneously. However, a 1-cm defect was identified between the right ureter and the ovarian ligament, confirming a right sciatic hernia (**Fig. 3A**). No additional inguinal or pelvic hernias were present. To obtain an adequate operative field, the right broad ligament and adnexa were suspended to the abdominal wall with traction sutures. The peritoneal incision was initiated at the point where the right external iliac vessels and the ovarian ligament intersect. The incision line was set approximately 3 cm ventral to the hernia orifice and then extended caudally for an additional 5 cm. The peritoneum was carefully dissected from the pelvic adipose tissue while avoiding injury to surrounding vessels and nerves. The hernia sac was completely dissected free, with confirmation that no adhesions was present between its dorsal side and the ureter. The ureter and surrounding adipose tissue were preserved toward the pelvic wall, and posterior dissection was extended approximately 3 cm beyond the orifice, with careful attention to the nerves entering the sciatic foramen (**Fig. 3B**). A self-fixating mesh (Pro Grip) was trimmed to fit the dissected area and to ensure at least a 3-cm overlap in all directions from the orifice. The mesh was inserted and placed over the dissected space (**Fig. 3C**). The peritoneum was then closed with a continuous absorbable suture (**Fig. 3D**). An adhesion barrier was applied to the closed peritoneum, and the procedure was completed. The operative time was 102 min, and blood loss was minimal.

### Outcome and follow-up

The postoperative course was uneventful, and the patient was discharged on POD 3. She has remained free of abdominal symptoms during outpatient follow-up, and the sciatic nerve-related pain in the right thigh has completely resolved.

## DISCUSSION

Sciatic hernia is classified as a pelvic hernia, along with obturator and perineal hernias, and is considered to be the rarest entity in this group. The condition occurs when pelvic organs protrude through a widened sciatic foramen due to weakening of the pelvic floor musculature, which may result from aging, trauma, malnutrition, or increased intra-abdominal pressure. It is characteristically observed in thin, old women.^1)^ The sciatic foramen is divided by the sacrospinous ligament into the cranial greater sciatic foramen and the caudal lesser sciatic foramen.^2)^ According to a recent review, approximately 90% of reported sciatic hernias occur through the greater sciatic foramen, whereas herniation through the lesser sciatic foramen accounts for less than 10% of cases.^3)^ Reported hernia contents include the small bowel, colon, ureter, ovary, bladder, and appendix, and the clinical presentation varies widely depending on the prolapsed organ.^4,5)^ In a review of 99 cases by Losanoff et al. in 2010, patients ranged from 5 weeks to 90 years of age, with women accounting for 77% of cases and bilateral involvement observed in 4%.^6)^ Presenting symptoms included abdominal pain in 50%, a palpable mass in 36%, bowel obstruction in 13%, and sciatic neuralgia in 8%.^6)^ In the present case, the hernia contained the small intestine; however, the patient had no abdominal pain, no signs of bowel obstruction, and no gastrointestinal symptoms attributable to small bowel prolapse. Instead, she had intermittent numbness in the right thigh for approximately 1 year, which was presumed to result from compression of the sciatic nerve by the herniated bowel. Sciatic hernias that arise through the greater sciatic foramen—particularly the suprapiriform foramen—may cause compression of the sciatic nerve, leading to thigh or buttock pain.^7)^ When small bowel prolapse progresses to incarceration, bowel obstruction may occur, and patients may present with abdominal pain, vomiting, or abdominal distension. In evaluating bowel obstruction in patients without prior abdominal surgery, it is important to assess the pelvis carefully and consider sciatic hernia alongside other pelvic and inguinal hernias.^8)^ For diagnosis, abdominal CT and pelvic MRI are currently the most useful imaging modalities. Urethrography may be useful in selected cases in which ureteral involvement is suspected. In addition, when contrast-enhanced CT cannot be performed, ultrasonography may help evaluate blood flow in the herniated organ. Pelvic MRI can provide detailed anatomical information on bones, muscles, and ligaments and, although interpretation may be challenging, may help assess the relationship between the herniated organ and nearby nerves as well as nerve changes caused by compression.^7)^ During imaging evaluation, determining whether the prolapsed organ shows signs of ischemia or injury is essential for surgical planning, particularly when deciding whether emergency surgery is required. In the present case, although small-bowel prolapse was identified at diagnosis, there were no findings suggestive of bowel obstruction or ischemia, and emergency surgery was therefore not indicated. Furthermore, confirming the presence or absence of other pelvic or inguinal hernias during imaging can help determine the optimal repair method. Notably, sciatic hernia is known to coexist with obturator hernia; one report found that 24% of patients diagnosed with obturator hernia on CT also had an asymptomatic sciatic hernia.^9)^ When an obturator hernia is present, repair including the obturator foramen should be considered. As in the present case, it is important to carefully assess intraoperatively not only for obturator hernia but also for other pelvic or inguinal hernias, and to perform simultaneous repair of any lesions that require surgical correction. Asymptomatic sciatic hernias do not necessarily require surgery; however, the decision to operate should consider symptoms, type of prolapsed organ, and impact on daily activities.^10)^ In this case, emergency surgery was not indicated; however, because the patient experienced right thigh numbness that impaired her QOL, elective surgical repair was appropriate. Treatment of sciatic hernia depends on hernia contents; however, the principles involve reduction of the herniated organ and closure of the orifice. Two surgical approaches are described: transabdominal and transgluteal. In cases such as ours, with small-bowel prolapse, the transabdominal approach is generally preferred. Laparoscopic surgery can be challenging when the small-bowel is incarcerated, as obstruction-related dilation can significantly impair visualization. However, recent reports suggest that laparoscopic repair is feasible in small-bowel prolapse without incarceration.^11)^ In this case, despite small-bowel prolapse, there were no signs of obstruction or ischemia, and sufficient time was available for preoperative evaluation, allowing laparoscopic repair. Various methods for closing the hernia orifice have been described, including primary suture closure, autologous tissue repair using structures such as the omentum, and mesh repair.^6)^ The choice among these techniques largely depends on the degree of contamination around the hernial orifice observed during surgery. In this case, because intraoperative contamination was unlikely, mesh repair was selected. Two general types of mesh are available: plug-type and sheet-type. Some reports describe using both; however, we chose only a sheet-type mesh because the patient had preoperative sciatic nerve-related pain, and placing a plug directly into the orifice could risk provoking or exacerbating sciatic neuralgia. No consensus exists regarding the optimal extent of dissection when placing a sheet-type mesh around the sciatic foramen. By analogy with conventional inguinal hernia repair using sheet mesh, we judged that a minimum overlap of 3 cm beyond the margins of the orifice was necessary to ensure adequate coverage. The ureter runs dorsal to the sciatic foramen, the infundibulopelvic ligament lies ventral, and the external iliac vessels course cranially. We therefore dissected within the area bounded by these structures to create a safe and sufficient space for mesh placement. Because the ureter can occasionally herniate through the sciatic foramen, placing the mesh on the pelvic-wall side of the ureter initially seemed reasonable. However, dissecting a normally positioned ureter away from the pelvic wall risks impairing peristalsis or blood supply. Ureteral herniation in sciatic hernia likely occurs when the peritoneum slides through the orifice, pulling the ureter along. For these reasons, we placed the mesh on the peritoneal side of the ureter. Future follow-up and additional cases will help determine whether a ureter in a normal anatomical course could subsequently prolapse through the sciatic foramen after mesh placement. Regarding mesh fixation, although some reports describe tacking or suture fixation around the orifice, the greater sciatic foramen contains the sciatic nerve, and unnecessary fixation may increase the risk of nerve injury. To minimize this risk, we avoided tacking or suturing and used a self-fixating mesh in this case. The sciatic foramen lies deep along the lateral pelvic wall, making visualization difficult, and the presence of critical structures such as the ureter and external iliac vessels necessitates meticulous surgical manipulation. In this case, we placed the patient in a steep Trendelenburg position to displace the small bowel cranially and suspended the infundibulopelvic ligament and ovary to the abdominal wall to secure an adequate operative field. These measures provided a sufficiently wide view of the deep pelvis, allowing the laparoscopic procedure to be performed safely. A PubMed search using the terms (“laparoscopic” OR “robot-assisted”) AND (“sciatic hernia” OR “ureterosciatic hernia”) AND (“mesh” OR “plug” OR “patch”) through October 2025 identified 10 previously reported cases in which sciatic hernia repair with mesh was clearly performed endoscopically, excluding the present case^7,11–19)^ (**Table 1**).

**Table 1:** Reported cases of laparoscopic mesh repair for sciatic hernia

Author	Year	Sex/Age	Side	Contents	Approach	Mesh type	Fixation
Gee et al.^12)^	1999	F/72	Left	Ureter	Laparoscopic	Not stated	Not stated
Witney-Smith et al.^13)^	2007	F/76	Left	Ureter	Laparoscopic	Plug	No
Bernard et al.^11)^	2010	F/67	Right	Small-bowel	Laparoscopic	Plug+patch	Yes
Whyburn et al.^14)^	2013	F/74	Bilateral	Ureter	Laparoscopic	Flat mesh	Not stated
Colombo et al.^15)^	2017	F/44	Right	Ovary	Laparoscopic	Flat mesh	No
Zeng et al.^16)^	2021	F/76	Bilateral	Small-bowel (right) + Sigmoid (left)	Robotic	Flat mesh	Yes
Van Hoef et al.^7)^	2021	F/82	Right	Small-bowel	Laparoscopic	Flat mesh	Yes
Fujimoto et al.^17)^	2022	F/81	Right	Small-bowel	Laparoscopic	Plug + patch	Not stated
Li et al.^18)^	2022	F/77	Right	Ureter	Laparoscopic	Flat mesh	No
Chihara et al.^19)^	2023	F/58	Right	Small-bowel	Laparoscopic	Flat mesh	No
Present case	2025	F/78	Right	Small-bowel	Laparoscopic	Flat mesh	No

F, female; Fixation, mesh fixation with sutures or tacks

Of the ten previously reported cases, nine represented primary sciatic hernias, and one was a recurrent case. In the recurrent case, the initial operation involved laparoscopic suture closure of the orifice without mesh reinforcement.^7)^ Nine cases were treated laparoscopically, and one was performed using a robot-assisted approach.^16)^ None of the patients had preoperative small-bowel obstruction, suggesting that the presence or absence of small-bowel dilatation may be a key factor in determining the suitability of a minimally invasive approach. The herniated organs included the small-bowel in four cases, the small-bowel and sigmoid colon in one case, the ureter in four cases, and the ovary in one case. Regarding laterality, six were right-sided, two were left-sided, and two were bilateral. Excluding two cases in which the mesh type was not specified, one used only a plug-type mesh, two used a combination of plug and patch, and the remaining five used a patch alone. Regarding mesh fixation, methods were not described in three cases; three used sutures or tacking, whereas four omitted mechanical fixation. Among the cases repaired with a patch, two explicitly quantified the extent of dissection, securing a 2–4 cm margin beyond the orifice before placing the mesh.^17,19)^ No postoperative recurrences were reported. Across these studies, meticulous dissection around the sciatic foramen—mindful of surrounding vessels, nerves, and the ureter—and careful consideration of mesh fixation were emphasized, with several cases omitting tacking or suturing. Regardless of the repair technique, long-term follow-up of more cases is needed to evaluate recurrence rates and postoperative complications. Because endoscopic repair of sciatic hernia remains rare worldwide, further accumulation of mid- and long-term outcomes is necessary, along with progress toward standardizing operative techniques.

## CONCLUSIONS

We report a case of sciatic hernia successfully repaired using a laparoscopic approach. Small-bowel prolapse was present; however, the absence of emergent findings allowed for elective surgery. During the procedure, tissues surrounding the hernia orifice were dissected, and the orifice was repaired using a self-fixating mesh. Despite our limited prior experience with this rare operation, careful preoperative imaging and meticulous efforts to secure a safe operative field enabled completion without complications. Further accumulation of mid- and long-term outcomes is needed to clarify the efficacy and durability of laparoscopic repair for sciatic hernia, including recurrence and late complications.

## DECLARATIONS

### Availability of data and materials

All data generated or analyzed during this study are included in this published article.
